# Management of a mixed ACTH- and prolactin-secreting pituitary adenoma during pregnancy

**DOI:** 10.1530/EDM-24-0094

**Published:** 2025-11-07

**Authors:** Astrid Le Rigoleur, Stefan Matei Constantinescu, Lina Daoud, Martin Lammens, Edward Fomekong, Frédéric Debiève, Dominique Maiter, Orsalia Alexopoulou

**Affiliations:** ^1^ Department of Endocrinology and Nutrition, Cliniques Universitaires Saint-Luc Brussels, Brussels, Belgium; ^2^ Department of Neuropathology, Cliniques Universitaires Saint-Luc Brussels, Brussels, Belgium; ^3^ Department of Pathology, Antwerp University Hospital, University of Antwerp, Antwerp, Belgium; ^4^ Department of Neurosurgery, Cliniques Universitaires Saint-Luc, Brussels, Belgium; ^5^ Department of Obstetrics, Cliniques Universitaires Saint-Luc, Brussels, Belgium

**Keywords:** pituitary, reproduction, rare diseases/syndromes, neuroendocrinology

## Abstract

**Summary:**

The diagnosis and management of Cushing’s disease (CD) during pregnancy are challenging. Only a few cases of mixed pituitary adenomas secreting prolactin and ACTH have been reported, and none during pregnancy. We report the case of a 30-year-old woman who presented with galactorrhea, weight gain, hypertension, prediabetes, dorsal fat pad, and abdominal striae. Initial biochemical investigations revealed hyperprolactinemia with increased ACTH but no biochemical signs of hypercortisolism. Pituitary MRI showed a 10 mm pituitary adenoma, which was first considered a prolactinoma potentially co-secreting ACTH. Surgery was indicated, but the patient did not undergo treatment immediately due to lack of health insurance. Cabergoline monotherapy was initiated, with close follow-up advised until regularization of social status in Belgium. The patient was then lost to follow-up and presented 15 months later because of an early pregnancy with treatment-resistant hypertension. Biochemical evaluation during the first trimester led to the suspicion of ACTH-dependent cortisol excess and showed hyperprolactinemia despite ongoing cabergoline treatment. She underwent transsphenoidal surgery at 16 weeks of pregnancy, and pathological examination showed a single adenoma with two different cell components staining for PRL/PIT1 and ACTH/TPIT, respectively. Surgery was successful, the patient developed corticotrope insufficiency, and was able to stop antihypertensive drugs. Because of failed induction of labor (for gestational insulin-requiring diabetes), she underwent cesarean section at 39 weeks of pregnancy and gave birth to a healthy boy with no maternal or neonatal complications. Adrenal insufficiency recovered 12 months after surgery. Genetic testing for MEN1 and AIP was negative.

**Learning points:**

- Mixed ACTH- and PRL-secreting pituitary adenomas, although extremely rare, do occur and may lead to both Cushing’s syndrome and galactorrhea/oligo- or amenorrhea syndrome. Cabergoline therapy is an option in such cases and may decrease both PRL and ACTH levels; however, its efficacy in controlling Cushing’s disease is limited.
- Preconception counseling is essential in women of childbearing age suffering from secreting pituitary tumors.
- Management of prolactinoma during pregnancy usually includes cessation of dopamine agonists and close follow-up to detect tumor growth, especially during the third trimester.
- Diagnosis of Cushing’s syndrome during pregnancy is challenging due to the physiological activation of the hypothalamic–pituitary–adrenal axis.
- Cushing’s syndrome may worsen during pregnancy and is associated with severe maternal and fetal complications. Early diagnosis and treatment are essential to improve fetomaternal outcomes.
- Transsphenoidal surgery performed during the second trimester is an effective and safe treatment for ACTH-secreting adenomas during pregnancy.

## Background

Prolactinomas in pregnant women are most often diagnosed before pregnancy, in the context of menstrual abnormalities, galactorrhea, and/or infertility, occurring in about 1/500 women of reproductive age. Medical treatment with dopamine agonists (DA) is indicated to normalize prolactin, decrease tumor size, and restore eugonadism (1). Once pregnancy is confirmed, DA can be safely stopped for microprolactinoma and for treated macroprolactinomas that have sufficiently decreased in size, but may be continued throughout pregnancy in women with a large prolactinoma that remains close to the optic chiasm (1, 2). The risk of prolactinoma growth without DA during pregnancy is about 10% in microprolactinomas and closer to 30% in macroprolactinomas (1). A surveillance MRI at the beginning of the third trimester is advised for large macroprolactinomas or tumors close to the optic chiasm (3), while serial prolactin measurements are not useful, as prolactin physiologically increases during pregnancy. Surgery is reserved for apoplexy or symptomatic tumor growth that does not respond to DA (1).

Pregnancy in patients with Cushing’s syndrome (CS) is rare due to the hypogonadism induced by cortisol excess. In the general population, CS has an annual incidence of 1.2–3.2 cases per million (4, 5). Only around 250 cases have been reported during pregnancy in the current literature (6). The predominant etiology of newly diagnosed CS in pregnant women is adrenal adenoma, whereas it is Cushing’s disease (CD) in nonpregnant women, probably because hypercortisolism and hyperandrogenism are generally more severe in CD, thus making the occurrence of pregnancy less likely (6). Moreover, women with active CS are advised not to become pregnant because of the increased risk of maternal and fetal complications (gestational diabetes, hypertension, preeclampsia, preterm delivery, and fetal loss) (1). Clinical manifestations during pregnancy include nonspecific signs such as weight gain, hypertension, facial plethora, and diabetes mellitus, but more specific signs such as ecchymosis, skin frailty, proximal myopathy, large purple striae, hypokalemia, and/or osteoporotic fractures may also occur (5). Its diagnosis during pregnancy is challenging because the hypothalamic–pituitary–adrenal (HPA) axis is physiologically activated in pregnant women (7). The preferred treatment of CD during pregnancy is pituitary surgery, though occasional use of metyrapone, ketoconazole, and cabergoline has been reported (1).

Only a few cases of mixed pituitary adenomas that secrete ACTH and prolactin have been reported, with some being responsive to cabergoline, but none have been described during pregnancy (8, 9, 10, 11). We report for the first time a patient with a mixed pituitary macroadenoma secreting ACTH and prolactin successfully treated by TSS during pregnancy.

## Case presentation

Our patient was born in Rwanda and arrived in Belgium when she was 25 years old. At age 26, because of oligomenorrhea, galactorrhea, headaches, and dizziness, she was diagnosed at another hospital with a centimetric pituitary prolactinoma (prolactin at diagnosis 82.9 μg/L (5.0–23.0 μg/L)). Treatment with cabergoline was initiated and titrated up to a dose of 1.5 mg/week, which was the maximum tolerated dose because of nausea and vomiting.

**Figure 1:**
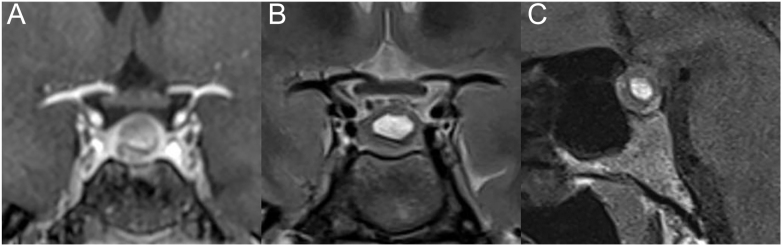
Pituitary MRI performed 10 months after diagnosis showing a stable 10 × 8 mm adenoma with necrotic hemorrhagic transformation and cystic center. (A) Post-gadolinium coronal T1-weighted image. (B) Coronal T2-weighted image. (C) Non-enhanced sagittal T1-weighted image.

One year after initiation of cabergoline, despite apparent good compliance by the patient, blood tests revealed persistent hyperprolactinemia (73.3 μg/L (5.0–23.0 μg/L)) and a significantly increased ACTH (three times the upper limit of normal) with normal cortisol levels. The follow-up MRI showed an adenoma with no change in size over 10 months but with a silent necrotic hemorrhagic transformation and no compression of the visual pathways (Fig. 1). No clinical signs of apoplexy were noted during follow-up.

Concurrently, CS was suspected because the patient had gained weight, presented striae, and developed prediabetes and high blood pressure. The patient was hospitalized in a regional hospital. Morning cortisol was 312.9 nmol/L, and morning ACTH was increased to 187.7 ng/L (4.7–48.8 ng/L). Evening cortisol was 214.7 nmol/L, and evening ACTH was 142.6 ng/L. However, a low-dose dexamethasone suppression test (DST) (0.5 mg every 6 h for 48 h) successfully suppressed plasma cortisol (26.6 nmol/L), and urinary free cortisol (UFC) was normal (34 μg/24 h (<40)). Because of financial constraints, late-night salivary cortisol was not measured, and corticotropin-releasing hormone (CRH) or desmopressin stimulation tests were not performed. IGF-1 was normal.

Surgical removal of the pituitary tumor was proposed, but this option was postponed until the patient could obtain sufficient medical insurance coverage. An increase in the cabergoline dose was not advised because of limited drug tolerance, financial issues, and the expectation of rapid neurosurgery. Close follow-up was therefore advised until administrative regularization with social services.

She was then lost to follow-up for 15 months and was referred urgently and for the first time to our endocrinology department because of the recent occurrence of a first spontaneous pregnancy (5 weeks’ amenorrhea) at the age of 29.

Her menstrual cycles had regularized with cabergoline, but galactorrhea persisted. She was taking metformin 1,500 mg/day for prediabetes and insulin resistance with polycystic ovary syndrome and alpha-methyldopa 1,000 mg/day for hypertension. She complained of headaches and asthenia. She had gained 50 kg in 4 years (BMI: 52 kg/m^2^). Her blood pressure was 150/90 mmHg. On clinical examination, she presented with dark abdominal stretch marks, buffalo neck, and lower limb edema. Acetylsalicylic acid (160 mg/day) was started to prevent eclampsia.

## Investigation

Hormonal evaluation before and during early pregnancy is shown in Table 1. Peripheral hypothyroidism was observed. Prolactin concentration measured at 8 weeks of gestation was stable at 73.3 μg/L on cabergoline treatment 1.5 mg/week, at a time when prolactin levels may be increased physiologically.

**Table 1:** Evolution of clinical and biological parameters during pregnancy and after neurosurgery.

	Normal range	15 months before pregnancy	Pre-surgery (8 weeks of pregnancy)	Post-surgery (17 weeks of pregnancy)	End of pregnancy (36 weeks of pregnancy)	6 months post-partum
Weight (kg)		130	147	152	159	150
BMI (kg/m^2^)		47	53	55	57	51
Blood pressure (mmHg)		170/110	150/90	125/75	130/70	120/70
Fasting glycemia (mg/dL)		83	79	106	/	93
HbA1c (%)		5.8	5.9	6	6.4	5.7
TSH (mU/L)	0.27–4.20	3.32	5.04	1.11	1.67	2.61
FT4 (pmol/L)	12.0–22.0	11.4	11.7	13.8	12.7	14.7
PRL (μg/L)	5.0–23.0	74.4	73.3	4.9	17.9	11.6
Morning cortisol (nmol/L)	130–500	312.9	417.4	86.4	/	249.9
ACTH (pg/mL)	5.0–49.0	187.7	187.5	9.1	14.5	53
UFC (μg/24 h)	<40.0	34	81.3	/	/	/

BMI, body mass index; TSH, thyroid-stimulating hormone; FT4, free T4; PRL, prolactin; ACTH, adrenocorticotropic hormone; UFC, urinary free cortisol.

**Figure 2:**
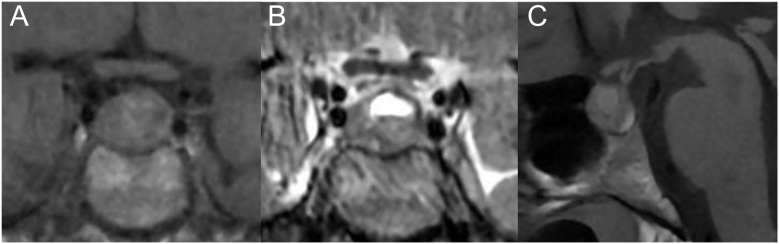
Pituitary MRI performed without contrast injection at 12 weeks of pregnancy showed overall stability of the adenoma. (A) Coronal T1-weighted image. (B) Coronal T2-weighted image. (C) Sagittal T1-weighted image.

Morning cortisol was normal at 417.4 mmol/L, but late afternoon cortisol was increased at 369 nmol/L, while ACTH levels remained high. UFC measured at week 8 was elevated for the time of pregnancy at 81.3 μg/24 h (measured by LC-MS/MS; normal value: < 40 μg/24 h). A pituitary MRI (without gadolinium injection), performed at 12 weeks of pregnancy, revealed overall stability of the adenoma, which measured 10 × 8 mm, with no compression of the visual pathways (Fig. 2). First-trimester fetal ultrasound was normal.

## Treatment

We discussed the patient’s case in a multidisciplinary meeting for pituitary tumors, with the presence of the obstetrical team. Due to the high risk of worsening of the CS during pregnancy and potential severe complications for the mother and the fetus, and due to the relative contraindications of anticortisolic drugs during pregnancy (1), neurosurgery during the second trimester was considered the best option for the patient. This choice was also influenced by the availability of an experienced pituitary neurosurgeon and the high likelihood of complete adenoma resection due to its midline localization. Of note, neurosurgery later in pregnancy would have exposed the patient to the risk of premature birth (12). Until neurosurgery, cabergoline was continued at the dose of 0.5 mg three times a week, and levothyroxine 75 μg/day was started for hypothyroidism. Steroidogenesis inhibitors were not considered as preoperative treatment. Ketoconazole, but not metyrapone or osilodrostat, is available in Belgium but is not approved for use in pregnant women.

**Figure 3:**
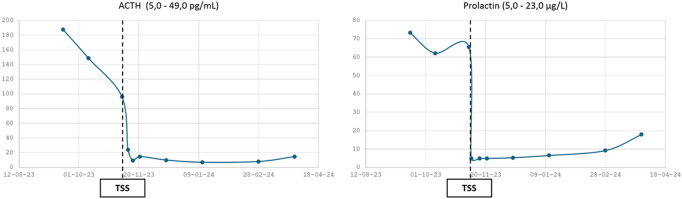
Evolution of plasma ACTH and serum prolactin concentrations during pregnancy. The dotted vertical line indicates the time of transsphenoidal surgery (TSS).

**Figure 4:**
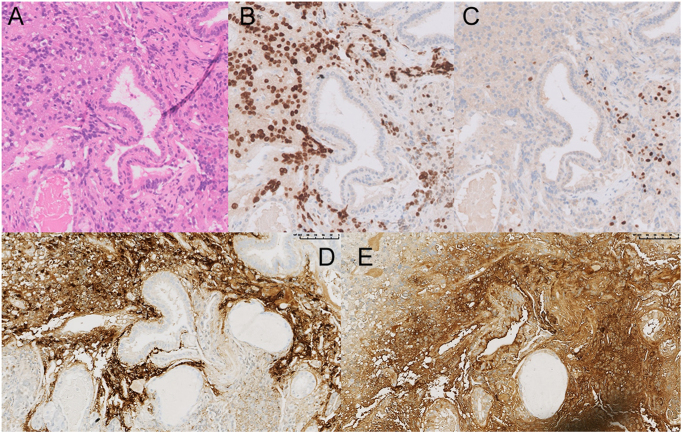
Pathological examination showed a single adenoma with mixed but separate PRL/PIT1 and ACTH/TPIT positive cell populations. (A) Hematoxylin and eosin stain. (B) PIT1 staining. (C) TPIT staining. (D) Prolactin staining. (E) ACTH staining. Sections (A), (B), and (C) are serial sections immediately adjacent to one another. Sections (D) and (E) are non-consecutive sections obtained from a more distant tissue plane.

The neurosurgeon performed TSS at 16 weeks of pregnancy without any complications. He removed a relatively well-encapsulated necrotic hemorrhagic lesion. The postoperative biological evaluation showed normalization of prolactin concentrations and the onset of corticotrope insufficiency (low morning cortisol and low-normal ACTH) (Table 1 and Fig. 3). Discharge treatment included methyldopa, hydrocortisone 20 mg in the morning, and 10 mg in the early afternoon, levothyroxine 75 μg/day, and thromboprophylaxis with low-molecular-weight heparin (LMWH) for 1 month (due to previous CD). Pathological examination showed a single adenoma with positive staining in different cellular components for PRL/PIT1 and ACTH/TPIT, respectively (Fig. 4). The PIT1-positive cells were negative for growth hormone and thyroid-stimulating hormone. Immunohistochemistry for prolactin and ACTH matched the expected transcription factors (Fig. 4).

## Outcome and follow-up

**Figure 5:**
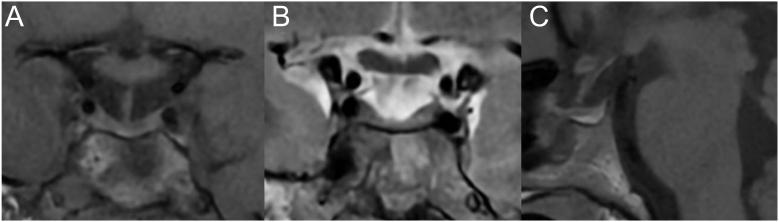
Postoperative pituitary MRI performed without contrast at 27, 5 weeks of pregnancy showing complete resection. (A) Coronal T1-weighted image. (B) Coronal T2-weighted image. (C) Sagittal T1-weighted image.

After surgery, we observed rapid normalization of blood pressure, and antihypertensive drugs could be stopped. Hba1c was 6.0% at the time of surgery and remained stable 1 month after surgery. Capillary glucose measurements performed at home remained in the target range until 24 weeks of pregnancy when insulin had to be started. At 20 weeks of gestation, we increased the doses of hydrocortisone to 30 mg in the morning and 20 mg in the early afternoon because of persistent fatigue, hypotension, and nausea. A pituitary MRI at 27 weeks of pregnancy showed complete tumor resection (Fig. 5).

Because of insulin-requiring gestational diabetes, the obstetricians scheduled labor induction at 38 weeks of pregnancy, but due to failure to induce labor, the patient underwent a cesarean section at 38 weeks and 6 days after injection of 100 mg hydrocortisone IV, with no complications. A healthy boy of 3.25 kg was born (Apgar score: 5-8-10). We doubled hydrocortisone doses for 48 h postpartum before reducing it to 20–10 mg at discharge. We stopped insulin postpartum and restarted metformin to reduce insulin resistance in an obese patient with preexisting PCOS.

The patient was unable to breastfeed because of low prolactin levels.

At 6 months postpartum, follow-up MRI showed no visible residual adenoma. An ACTH stimulation test demonstrated recovery of the corticotrope axis, allowing hydrocortisone to be discontinued. Prolactin levels, without dopamine agonist treatment, were normal at 11.5 μg/L. Glycemic control was adequate with metformin (HbA1c: 5.7%), and blood pressure was normal without treatment. However, obesity persisted (BMI: 51 kg/m^2^), and the patient is currently undergoing evaluation for bariatric surgery.

Genetic testing for *MEN1* and *AIP* was performed and showed the absence of pathogenic mutations.

## Discussion

We report the case of a young woman presenting with a plurihormonal pituitary macroadenoma first secreting prolactin and leading to a galactorrhea/oligomenorrhea syndrome, which was treated with dopamine agonists, and later inducing an overt ACTH-dependent Cushing syndrome diagnosed during early pregnancy. The patient could be successfully treated by TSS during the second trimester.

Only a handful of mixed ACTH-PRL secreting adenomas have been reported (8, 9, 10, 11, 13), two of which had genetic predisposition (MEN1 in one case (10) and AIP mutation in another (13)). This association of hormones in the same tumor is unusual because of the different cell lineages and transcription factors necessary for the differentiation of lactotroph (PIT-1 dependent (14)) and corticotroph cells (T-PIT dependent (15)). We show, however, that although both hormones came from the same tumor, two distinct cellular populations (lactotroph and corticotroph) were present and remained separate inside the adenoma. In other case reports of ACTH-PRL secreting adenomas, immunohistochemistry has shown that adenoma cells can either be positive for both ACTH and PRL (10) or consist of two distinct cell populations that are intermingled (9).

Diagnosis of CS is challenging during pregnancy, as symptoms can be mistaken for symptoms of healthy or complicated pregnancy, and because the HPA axis is progressively activated in normal conditions. UFC is broadly similar to non-pregnant levels during the first trimester but increases to 2–3 times in the 2nd and 3rd trimesters (7). Low-dose DST fails to suppress plasma cortisol (7). The circadian rhythm is preserved but with less relative variation, and late-night salivary cortisol is about two times higher (7). In the present case, despite elevated ACTH levels, the biological (though restricted) work-up for the diagnosis of hypercortisolism was negative before pregnancy (normal dexamethasone test and normal UFC). We thus hypothesize that the adenoma was first silently producing ACTH without inducing excessive cortisol production, at least at the time of the work-up performed 1 year before pregnancy. High ACTH with normal cortisol secretion (including normal biological work-up for hypercortisolism) is a classical feature of silent corticotroph adenomas (SCA) (16). The mechanisms involve the secretion of less active or inactive forms of ACTH or POMC (17).

At the time of pregnancy diagnosis, there was evidence that the patient had progressed from SCA to moderate overt Cushing disease, without knowledge of the exact timing of this progression. Indeed, in a context of clinical manifestations suggestive of hypercortisolism (abdominal striae, dorsal fat pad, worsening hypertension) and persistently elevated ACTH levels, the diagnosis of overt CD was based on the loss of a normal cortisol circadian rhythm and a two-fold increase in UFC at the beginning of pregnancy, at a time when UFC is not yet significantly increased (7). The diagnosis of a plurihormonal PRL-ACTH containing adenoma was ultimately confirmed by pituitary pathology with both positive PRL/PIT1 and ACTH/T-PIT immunostaining in different cells, while the diagnosis of overt CS was confirmed by the development of immediate postoperative corticotropic deficiency that recovered 6 months postpartum. Of note, such a progression from SCA to overt CD has been reported in up to 9% of cases (18).

Some authors have speculated that Cushing syndrome may worsen or be revealed during pregnancy because of the placental CRH and ACTH production (19), but also because of the estrogen-induced increase in CBG, which reduces the negative feedback on ACTH secretion (7). The cortisol excess, however, remained moderate and contrasted with the persistently high ACTH levels, thus suggesting the persistent secretion of less bioactive forms of ACTH.

Sixty-three published cases of Cushing disease during pregnancy were recently reviewed (20), only 13 of which had surgery during pregnancy. Among this group of 13 patients, the most common symptoms were hypertension, obesity, hirsutism, and diabetes mellitus. The authors noted that the work-up consisted of 24 h UFC and ACTH/cortisol measurements in all, but only one patient had LNSC measured. Histology was positive for ACTH in all, and 10/13 entered complete remission, with one partial remission.

Cabergoline has shown some efficacy in case reports of ACTH-PRL secreting pituitary tumors, with decreases in both PRL and ACTH levels (8, 10, 11). We did not consider it likely that cabergoline would have sufficient beneficial effects on ACTH secretion in our case, as ACTH and prolactin levels were still elevated despite medical treatment at relatively high doses. Moreover, the efficacy of cabergoline as a treatment for CD is usually low, although an excellent response with recovery of eucortisolism has been reported in a case of PRL/ACTH co-secreting adenoma (13). If our patient had not developed some degree of hypercortisolism, we would have stopped cabergoline and performed an MRI in the third trimester, reserving surgery for symptomatic growth.

Occurrence of pregnancy in a patient with simultaneous lactotroph and corticotroph pituitary tumor is particularly surprising. We suppose this was possible because of the regularization of her menstrual cycles with DA and because clinical hypercortisolism was absent or very mild before pregnancy started. Preconceptional counseling is essential in women of childbearing age with CD (1). Ideally, our patient should have been on contraception until a good control of her secretory pituitary tumor was obtained.

## Declaration of interest

The authors declare that there is no conflict of interest that could be perceived as prejudicing the impartiality of the work reported.

## Funding

SMC is a recipient of a Doctoral Fellowship of the Belgian Fonds National de la Recherche Scientifique (reference Specialiste Doctorant 40011958).

## Patient consent

Written informed consent for publication of clinical details and/or clinical images was obtained from the patient.

## Author contribution statement

ALR wrote the first draft. SMC supervised the writing of the first draft and was responsible for endocrine care. EF was the operating neurosurgeon and revised the manuscript. ML and LD were responsible for pathological analysis and revised the manuscript. FD was responsible for obstetrical management and revised the manuscript. DM provided advice for endocrine treatment during pregnancy and revised the manuscript. OA supervised endocrine treatment and follow-up during pregnancy and revised the final manuscript.
